# A Non-Targeted Capillary Electrophoresis-Mass Spectrometry Strategy to Study Metabolic Differences in an In Vitro Model of High-Glucose Induced Changes in Human Proximal Tubular HK-2 Cells

**DOI:** 10.3390/molecules25030512

**Published:** 2020-01-24

**Authors:** Samuel Bernardo-Bermejo, Elena Sánchez-López, María Castro-Puyana, Selma Benito-Martínez, Francisco Javier Lucio-Cazaña, María Luisa Marina

**Affiliations:** 1 Departamento de Química Analítica, Química Física e Ingeniería Química, Universidad de Alcalá, Ctra. Madrid-Barcelona Km. 33.600, Alcalá de Henares, 28871 Madrid, Spain; samuel.bernardo@edu.uah.es (S.B.-B.); elena.sanchezl@edu.uah.es (E.S.-L.); maria.castrop@uah.es (M.C.-P.); 2 Instituto de Investigación Química Andrés M. del Río (IQAR), Universidad de Alcalá, Ctra. Madrid-Barcelona Km. 33.600, Alcalá de Henares, 28871 Madrid, Spain; 3 Departamento de Biología de Sistemas, Universidad de Alcalá, Ctra. Madrid-Barcelona Km. 33.600, Alcalá de Henares, 28871 Madrid, Spain; selma.benito@uah.es (S.B.-M.); javier.lucio@uah.es (F.J.L.-C.); 4 “Ramón y Cajal” Health Research Institute (IRYCIS), Universidad de Alcalá, 28871 Madrid, Spain

**Keywords:** diabetic nephropathy, human proximal tubular HK-2 cells, capillary electrophoresis-mass spectrometry, metabolomics, multivariate analysis

## Abstract

Diabetic nephropathy is characterized by the chronic loss of kidney function due to high glucose renal levels. HK-2 proximal tubular cells are good candidates to study this disease. The aim of this work was to study an in vitro model of high glucose-induced metabolic alterations in HK-2 cells to contribute to the pathogenesis of this diabetic complication. An untargeted metabolomics strategy based on CE-MS was developed to find metabolites affected under high glucose conditions. Intracellular and extracellular fluids from HK-2 cells treated with 25 mM glucose (high glucose group), with 5.5 mM glucose (normal glucose group), and with 5.5 mM glucose and 19.5 mM mannitol (osmotic control group) were analyzed. The main changes induced by high glucose were found in the extracellular medium where increased levels of four amino acids were detected. Three of them (alanine, proline, and glutamic acid) were exported from HK-2 cells to the extracellular medium. Other affected metabolites include Amadori products and cysteine, which are more likely cause and consequence, respectively, of the oxidative stress induced by high glucose in HK-2 cells. The developed CE-MS platform provides valuable insight into high glucose-induced metabolic alterations in proximal tubular cells and allows identifying discriminative molecules of diabetic nephropathy.

## 1. Introduction

The International Diabetes Federation reported in 2017 that 451 million people have diabetes mellitus and estimated 5 million deaths worldwide as a result of its complications. In addition, the number of patients expected in 2045 is 693 million [1]. This fact means that this disease is considered one of the most important public health issues of the twenty-first century by the World Health Organization (WHO). According to WHO, diabetes mellitus encompasses metabolic alterations of several etiologies characterized by chronic hyperglycemia and disorders in the metabolism of proteins, carbohydrates, and fats, due to the defects in insulin secretion [2]. It can develop multiple acute and chronic complications, which implies a high health risk. Among these complications, diabetic nephropathy is an important chronic kidney disease causing end-stage renal failure globally, which has no known cure and it can be clinically characterized by proteinuria, albuminuria, high creatinine levels, and irregular glomerular filtration rates [3]. To find new strategies for the prevention, early detection, or the study of diabetic nephropathy progress, it is necessary to know the discriminative molecules in order to identify the molecular mechanisms involved in this disease [4]. In this way, metabolomics, especially in the non-targeted mode, offers great possibilities for studying the metabolome and the mechanisms implicated using different analytical techniques such as liquid chromatography-mass spectrometry (LC-MS), gas chromatography-mass spectrometry (GC-MS), or capillary electrophoresis-mass spectrometry (CE-MS).

Although cell metabolomics is less frequently used than other biological samples, it is very useful to study different pathologies and to improve the knowledge about them [5]. Likewise, the use of in vitro model cells allows obtaining results, which are easily interpreted especially in the case of cell cultures without taking into account factors such as age or gender, among others, except primary cultures [5]. Studies have shown that the renal proximal tubule is the principal mover in diabetic kidney pathology and has an important role in the different phases of this disease [6]. Thus, the human proximal tubular HK-2 cell line is a good candidate for studying how high glucose-induced changes contribute to the genesis and progression of diabetic nephropathy. In fact, our research group has previously performed the first untargeted metabolomics analysis of HK-2 cells, which enabled us to obtain relevant information about diabetic nephropathy using LC-MS [7]. In addition, So et al. carried out a study about diabetes on HK-2 cells using a proteomic approach [8]. These cells were cultivated with different glucose concentrations and, when high glucose level was compared to a normal glucose level, five proteins (three of them related to oxidation of glucose) showed statistically significant differences. In this way, this work provided an overview of protein targets of stress by high glucose in HK-2 cells.

LC-MS and GC-MS are the most frequent analytical techniques employed in a wide variety of targeted and non-targeted metabolomics studies of different samples. However, the use of CE-MS for metabolomics analysis has been increasing in recent years [9]. This technique is considered a great complementary tool for increasing the coverage of the metabolome obtained by the previously mentioned analytical techniques [10]. The main limitation is the flow-rate employed in CE, which is in the nL/min order. This makes it necessary to use a sheath liquid to ensure the electrospray formation. This additional flow dilutes the samples considerably due to the flow-rate of sheath liquid being µL/min. Currently, the development of interfaces as the sheathless interface improve the sensitivity in the metabolomic analysis [9]. Nonetheless, CE-MS is a good alternative and has proved to be useful in the analysis of polar and charged metabolites in biological samples (urine, plasma, serum, or tissues) and endogenous compounds, such as nucleotides and amino acids [11]. For this reason, CE-MS is considered an orthogonal technique to LC-MS, which requires minute amounts of the sample [11].

Recently, Wei et al. have carried out a metabolomic study of HK-2 cells using a CE-MS platform [12]. Different experimental groups of HK-2 cells were treated with glucose and mannitol at 5 mM or 25 mM in order to find characteristic changes in the metabolic profiles in response to hyperglycemia. The results concluded an increase in lactate-to-pyruvate ratio and cellular methylation potential, and a reduction in the metabolites involved in the Krebs cycle and glutathione antioxidant activity. However, this study only focused on the analysis of intracellular fluid, which can be considered a limitation since the information provided by extracellular fluid gives information related to the metabolites excreted and taken up by the cells. In addition, this work did not describe any methodological development since the CE-MS analysis was based on methods previously reported in the literature, which were validated for other type of samples such as Bacillus [13,14] and soy sauce [15] but not specifically for HK-2 cells.

The aim of this work was to develop a non-targeted metabolomics CE-MS platform for finding the affected metabolites of diabetic nephropathy through the analysis of an in vitro model of high glucose-induced metabolic alterations in HK-2 cells. Different variables were optimized, such as the extraction solvent, running buffer composition, and other parameters affecting CE-MS analysis (sheath liquid composition and flow, and drying gas flow). Then, both endo-metabolomes and exo-metabolomes were studied in a comprehensive way, analyzing both intracellular and extracellular fluids in order to expand the metabolic coverage and to obtain the maximum information from these cells. The main changes induced by high glucose were found in the extracellular medium in which we detected increased levels of four amino acids. Three of them (i.e., alanine, proline, and glutamic acid) were exported from HK-2 cells to the extra-cellular medium. Proline deserves further study because the potential role of d-proline as a biomarker of the progression of diabetic nephropathy [16] and because there are not previously described proline export transporters in the proximal tubule. Other metabolites identified in the extracellular medium were Amadori products (i.e., *N*-(1-Deoxy-1-fructosyl)leucine or *N*-(1-Deoxy-1-fructosyl)isoleucine and *N*-(1-Deoxy-1-fructosyl)phenylalanine) and cysteine, which more likely are cause and consequence, respectively, of the oxidative stress induced by high glucose in proximal tubular cells [17]. Regarding the intracellular changes in metabolites, there was a decrease in arginine that might be pathologically relevant because blocking its arginase-2-dependent degradation prevents the development of diabetic nephropathy [18]. The present article represents the first non-targeted metabolomics work focused on the analysis of both fluids in HK-2 cells by CE-MS.

Chronic hyperglycemia is considered to play a major role in the pathogenesis of diabetic kidney disease so that inadequate metabolic control over many years might lead to this pathology [19]. Accordingly, the diabetes model based upon exposure of cultured proximal tubular cells to high glucose concentrations is widely used to explore the mechanisms involved in diabetic nephropathy. In this context, our work highlights that the use of a non-targeted metabolomics CE-MS platform provides insight into high glucose-induced metabolic alterations in proximal tubular cells as well as allows the identification of discriminative molecules of diabetic nephropathy.

## 2. Results and Discussion

### 2.1. Optimization of the Sample Treatment and Analysis by CE-MS

The purpose of this work was to develop a non-targeted metabolomic platform based on CE-MS to study diabetic nephropathy using an in vitro model of high glucose using human proximal tubular cells HK-2. In order to obtain the largest number of molecular features and the best performance, the steps related to sample treatment and sample analysis were optimized. Both the intracellular and extracellular fluids were taken into account for analysis to expand the output obtained given the high complementarity of both approaches in the study of the cell metabolome. The initial CE-MS working conditions were selected from the article of Kuehnbaum et al. [20]. An uncoated fused-silica capillary of 50 µm ID with a total length of 110 cm was employed, the running buffer was 1 M formic acid containing 15% (*v/v*) acetonitrile, 30 kV was applied with 35 min of total analysis, and the temperature was set at 25 °C. The sheath liquid composition was methanol/water 60:40 (*v/v*) containing 0.1% (*v/v*) of formic acid and 0.02% (*v/v*) of purine and HP-921 and the flow rate was 6 µL/min. Then, these experimental conditions were, subsequently, optimized using the intracellular fluid of HK-2 cell samples as the model. The first step was to select the most appropriate extraction conditions to obtain the largest number of molecular features. Since large molecules such as proteins might disrupt the electroosmotic flow by sticking to the capillary walls, it was necessary to work with high organic content. Thus, acetonitrile and methanol at 75% (*v/v*) in water were compared. The best solvent was methanol because a high number of molecular features was observed. In order to find the best amount of organic solvent, 75% methanol was compared to an even higher solvent content, i.e., 100% methanol. In this sense, 60 molecular features were obtained using 75% methanol and 66 features were obtained using 100% methanol. Therefore, 100% methanol was selected.

Once the extraction conditions were selected, the running buffer composition was optimized. As can be seen in previous metabolomics studies based on CE-MS, 1 M formic acid is the running buffer of choice in cationic detection due to its lower current (under 50 µA) to improve ion signal stability [9]. In some cases, the presence of low percentages of organic solvent in the running buffer, e.g., acetonitrile, has also shown to improve peak efficiency [21]. However, the prolonged contact of the capillary with the organic solvents in the running buffer can deteriorate the end of the fused-silica capillary, which causes frequent problems such as the capillary fracture [22]. Thus, the effect of acetonitrile in the running buffer was investigated using 1 M formic acid and 1 M formic acid containing 15% (*v/v*) of acetonitrile. The number of components was 61 in 1 M formic acid and 53 in 1 M formic acid containing 15% (*v/v*) of acetonitrile, which is the RSD of the migration time of the IS lower in the first case (0.59% vs. 0.67%). For this reason, 1 M formic acid without acetonitrile was the running buffer selected.

The next step studied was the possibility to apply an additional pressure in combination with the separation voltage during the sample analysis. Some authors have reported that applying pressure reduces migration times and increases the effective mobility measurements and the repeatability of the migration time in the analyses [23]. Thus, analyses were carried out using 25 mbar and, without applying pressure, the results were compared. A total of 66 molecular features were obtained when applying 25 mbar and 41 features were obtained when the pressure was not applied. Additionally, the RSD of the migration time of the IS was lower when the pressure was used (0.65% vs. 1.04%). Therefore, 25 mbar pressure was employed in the analyses.

Next, the sheath liquid composition was optimized. The coaxial sheath liquid interface is the most frequently used for coupling CE to MS in metabolomics analyses [9] due to the fact that it is more stable, repeatable, and cost effective [24]. The two mixtures of organic solvent-water most usually employed are methanol and isopropanol in different percentages [25]. Thus, these two solvents were compared using a composition of 60:40 (*v/v*) organic solvent/water. The mixture isopropanol/water was chosen because it showed about a two times higher number of molecular features than methanol/water (123 vs. 62). In addition, it has been demonstrated that the mixture isopropanol/water allows the obtainment of a stable MS signal as a consequence of its good conductivity and volatility [26]. In order to find the best isopropanol/water proportion, three different percentages (60:40, 50:50, and 80:20 (*v/v*)) were evaluated. It was observed that, when the amount of organic solvent was the highest (80:20 *v/v*), the CE-MS current was not stable. Among the other two percentages tested, 60:40 *v/v* was selected not only based on the number of molecular features (105 vs. 98) but also because the signals were more intense. Additionally, the flow rate of the sheath liquid was optimized since, along with the composition of the sheath, liquid has an important effect on the CE-MS analysis [27]. The number of molecular features observed were 67, 87, and 95 when using 4, 6, and 8 µL/min, respectively, so that the highest flow rate was selected. It is worth highlighting that, using a relatively high flow rate, the sensitivity of the analysis increased (Figure S1).

Lastly, the flow rate of the drying gas of the ESI source was studied since it is an important factor, which helps the buffer desolvation and facilitates spray formation and the nebulization [28,29]. The drying gas also enhances the sensitivity [30]. The flows evaluated were 6, 8, and 10 L/min and the number of molecular features obtained under each condition was 106, 103, and 72, respectively, so that 6 L/min was selected. The drying gas also affects the sensitivity. Previous studies have shown that flow rates higher than 6 L/min can reduce the peak height in MS [30].

Once the CE-MS experimental conditions were optimized using the intracellular fluid as a model sample, these conditions were employed to perform the CE-MS analysis of the extracellular fluid with the aim of optimizing the extraction step. Methanol and acetonitrile were compared as extracting solvents. The first one was chosen because it enabled us to obtain a higher number of features (140) than when acetonitrile was used (69). Moreover, the signals of the total compound electropherogram (TCE) were more intense (see Figure S2).

### 2.2. Non-Targeted Metabolomics Analysis of An in Vitro Model of High Glucose in HK-2 Cells

This study was based on the analysis of intracellular and extracellular fluids of three different groups of HK-2 cells: NG group (cells treated with 5.5 mM d-glucose), HG group (cells treated with 25.0 mM d-glucose), and M group (cells treated with 5.5 mM of d-glucose and 19.5 mM mannitol). The last group was established in order to control changes in the metabolome due to osmotic pressure caused by high glucose content. This in vitro model has been used in previous reports [31].

Two metabolomics sequences were carried out in order to analyze both intracellular and extracellular fluids. These two sequences were constituted by six biological and three instrumental replicates of each group of samples (i.e., 54 samples). In addition, a total of 15 injections of the quality control (QC) samples were distributed during the sequence (see Section 3.5). Data obtained from the two sequences were treated according to Section 3.6. Lastly, after molecular feature alignment and filtering, the resulting datasets consisted of 59 and 100 variables for intracellular fluid and extracellular fluid, respectively.

Due to the complexity of the generated datasets in metabolomics studies, it is necessary to resort to multivariate statistical analysis. First, unsupervised principal components analysis (PCA) was employed to observe the possible significant metabolic differences among the three groups of samples and to assess the consistency of the two sequences with the QC samples clustering. In the PCA score plots, observations (i.e., samples) are represented in a two-dimensional plot, in which the axis display the two principal components representing most of the variability covered by the model. Samples that remain close together indicate that they have similar metabolic profiles. Data were normalized using the area of methionine sulfone, i.e., the internal standard (IS). Methionine sulfone was chosen over tyramine because the QC samples were better clustered together when using IS as a normalization peak, which is shown in Figure S3. In metabolomics studies by CE-MS, correction to IS is common because of larger variability inherent to this technique [32,33]. Data were further normalized to the protein content as in previous contributions [7,34,35].

**Figure 1:**
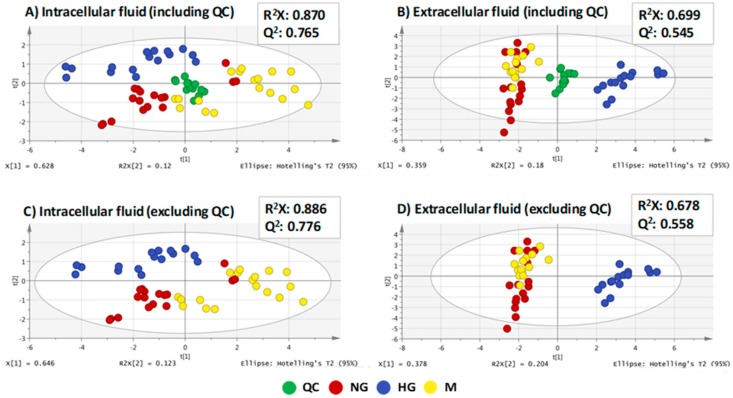
PCA including QC and excluding QC for the two analytical sequences: (**A**) intracellular fluid (including QC), (**B**) extracellular fluid (including QC), (**C**) intracellular fluid (excluding QC), and (**D**) extracellular fluid (excluding QC). QC, quality control. NG, normal glucose. HG, high glucose. M, osmotic control.

The two metabolomics sequences were performed successfully, as can be seen by the good clustering observed in the QC samples. Additionally, the models showed to be robust because the PCA models excluding the QC samples were comparable to the PCA, which included the QC samples (Figure 1). This means that the model was not affected by the presence of the QC samples in the plot. This was supported by hierarchical cluster analysis (HCA) (see Figure S4).

In the intracellular fluid, there was a clearer difference among the three groups of samples than in the extracellular fluid, and the R^2^X and Q^2^ values were higher as well. In the extracellular fluid, there was better clustering between the different groups of the samples. However, in this case, there were less significant differences between the NG group and M group based on their clustering.

Next, partial least square discriminant analysis (PLS-DA) models were built comparing, first, HG and NG groups and then NG and M groups in the two sequences in order to find the most important differences between the experimental groups. Unlike PCA, PLS-DA is a supervised method, which means that it uses information on experimental groups provided. These models are a combination of regression and classification techniques that reduces the dimensionality of the system variables, using partial least squares. In both cases, the PLS-DA models for intracellular fluid showed R^2^X, R^2^Y, and Q^2^ parameters higher than extracellular fluid models.

**Figure 2:**
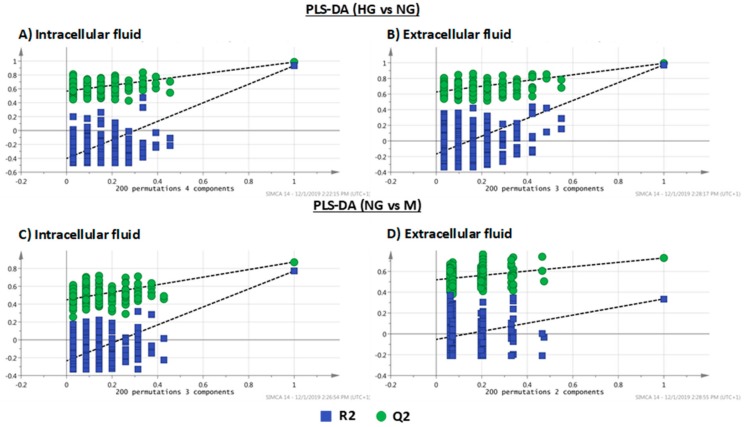
Permutation tests of PLS-DA models of both intracellular and extracellular fluids: (**A**) intracellular fluid (HG vs. NG), (**B**) extracellular fluid (HG vs. NG), (**C**) intracellular fluid (NG vs. M), (**D**) extracellular fluid (NG vs. M).

PLS-DA has the risk of having overfitting, i.e., that sample group separation is observed even though there are no differences in their metabolic profiles. To make sure that the separation provided by PLS-DA is real, it is necessary to validate these models. Thus, cross validated ANOVA (CV-ANOVA) was conducted for these models and F- and *p*-values were calculated. In general, the models showed high F-values and low *p*-values except in the PLS-DA model NG vs. M of extracellular fluid. All parameters are included in Table 1. Another way to double check the validity of PLS-DA models is conducting permutation tests. The permutation test based on 200 permutations was carried out for validating all PLS-DA models (Figure 2). As can be seen by the positive slopes found in all plots, there is no overfit existing, which validates, once again, the proposed models.

In order to find discriminative molecules, the molecular features that changed significantly between HG vs. NG were selected. Additionally, the molecular features that varied significantly between both HG vs. NG and NG vs. M, simultaneously, were selected because these changes could be due to a poorly controlled diabetes. For this reason, molecular features with a *p*-value (calculated using the Mann-Whitney U test) lower than 0.04 only in HG vs. NG and variables with a *p*-value lower than 0.04 in HG vs. NG and NG vs. M were selected. The threshold of 0.04 was calculated after the Benjamini-Hochberg correction for multiple testing.

### 2.3. Identification of Affected Metabolites

After data processing, a total of 13 and 34 variables were selected as statistically significant features from intracellular and extracellular fluids of HK-2 cells, respectively (see Table 2 and Table S1). The identification of these molecular features was carried out using the tools described in Section 3.7. In this way, 10 molecular features were identified as discriminative molecules of diabetic nephropathy in the fluids analyzed in this work. Table 2 shows the migration time, the molecular formula, the experimental *m/z* (Da), the mass error (ppm), the main MS/MS fragments in order of abundancy, the corrected *p*-values from the univariate Mann Whitney U test, and the trend for each identified metabolite. Arginine and pyroglutamic acid were unequivocally identified in intracellular fluid comparing the migration times and MS/MS fragmentation spectra with those of the standards. In the same way, five metabolites (histidine, alanine, proline, cystine, and tyrosine) were also identified in extracellular fluid. Moreover, glutamic acid was unequivocally identified as a relevant metabolite in both intra and extracellular fluids. The Amadori compounds *N*-(1-Deoxy-1-fructosyl)leucine or *N*-(1-Deoxy-1-fructosyl)isoleucine and *N*-(1-Deoxy-1-fructosyl)phenylalanine were tentatively identified comparing their MS/MS fragmentation spectra with those found in the literature [36]. Table S1 includes the information of the molecular features whose identity could not be verified.

### 2.4. Biological Interpretation of the Results

It is well known that the kidney plays an important role in the glucose homeostasis using several processes such as filtration, reabsorption, and consumption of glucose. In addition, gluconeogenesis also takes place in the proximal tubule [37].

In Section 2.3, a total of 10 different metabolites were identified either unequivocally or tentatively in both fluids and Figures S5–S7 show the box-plots displaying their levels in the analyzed samples.

In the intracellular fluid, the concentrations of two metabolites, arginine and pyroglutamic acid, were negatively and slightly positively affected by high glucose, respectively. Arginine is one of the most versatile amino acids because it serves as a precursor for the cell synthesis of nitric oxide, creatine, polyamines, urea, proline, glutamate, and proteins [38]. In our experimental context, it is particularly relevant that arginine is metabolized by the nitric oxide synthase (NOS) pathway and the arginase pathway [39], which are known to be increased in animal models of diabetes. In the streptozotocin rat model of type I diabetes, neuronal and endothelial NOS expression has been found increased in the proximal straight tubules [40]. Therefore, one may hypothesize that an increase in NOS activity is responsible for the decreased content in arginine found in HK-2 cells under high glucose conditions. However, specific experiments should be performed to confirm this hypothesis. In animal models of diabetic nephropathy, arginase-2 (which is the predominant renal isoform [41]) is also increased, so that blocking arginase-2 activity or expression prevents the development of diabetic nephropathy [18]. Since arginase hydrolyses l-arginine, the decreased content in l-arginine we have found in proximal tubular HK-2 cells exposed to high glucose might be pathologically relevant because it might be due to increased arginase-2 activity. The other metabolite affected by high glucose in the intracellular fluid was pyroglutamic acid, which is a product of amino acid catabolism, which was also identified in our previous metabolomics study in HK-2 cells by LC-MS [7].

In the extracellular fluid, seven different metabolites increasing in this study were identified. The increase in histidine in the extracellular medium from high glucose -exposed HK-2 cells may be due to its reduced metabolic degradation but also to its reduced uptake from the culture medium (histidine is present in its formulation).

Histidine is the precursor of histamine produced in human proximal tubules [42] and whose vasoactive and inflammatory properties have been suggested to have an important role in the early phase of diabetic nephropathy [43]. In this context, our results suggest the idea that high glucose may induce a histidine-dependent increase in the release of histamine by proximal tubular cells, which contributes to the genesis and/or progression of diabetic nephropathy. Worth mentioning, the urinary excretion of histidine is decreased in a model of type I diabetes by injecting streptozotocin in male rhesus monkeys [44], which suggests that other factors in addition to the handling of histidine in the proximal tubule are responsible for the final concentrations of amino acids found in urine.

Alanine, which is another metabolite identified in extracellular fluid, is one of the most abundant essential amino acids in the circulation [45] and it has an important role in the human body. Stancáková et al. carried out a study with humans and concluded that alanine levels increased with increasing glycemia in urine [46]. Alanine is not present in the formulation of high glucose medium and, therefore, it must have been transported from the intracellular compartment to the extracellular fluid. The SLC7A9 antiporter may deliver alanine (and other neutral amino acids) into the extracellular compartment in exchange for glutamate and cystine [47] and, therefore, it is theoretically possible that SLC7A9 activity may explain the higher content in alanine found in the extracellular fluid from HK-2 cells exposed to high glucose. Once alanine reaches the extracellular compartment, it may be taken up again (following a mechanism driven by the Na^+^-electrochemical gradient) by the SLC6A19 transporter, which is responsible for the recovery from the glomerular filtrate of alanine and other neutral amino acids [47]. However, it has been described that d-glucose inhibits alanine uptake [48], which may also contribute to the higher content in alanine found in the extracellular fluid from HK-2 cells exposed to high glucose.

Proline is an essential hydrophobic amino acid that plays important roles in several cell signaling pathways. Proline is not present in the formulation of high glucose medium and, therefore, it must have been transported from the intracellular compartment to the extracellular fluid. However, in the kidney, only transporters involved in proline uptake have been described [49]. Therefore, our results suggest that an unknown transporter is responsible for the increased content in proline in the extracellular fluid from HK-2 cells exposed to high glucose. Another related issue is the involvement of d-proline in our results. Although cells specifically synthesize the L isomers of amino acids, d-amino acids also exist in humans in trace amounts [16] and they are being increasingly considered as discriminative molecules. It is possible that an increase in d-proline may contribute to the higher levels of proline in the extracellular fluid from HK-2 cells exposed to high glucose because i) it has been recently found that serum levels of d-proline are significantly associated with the progression of diabetic nephropathy [16] and ii) kidney tubules handle the metabolism of d-amino acids [50]. However, further studies are required to confirm this hypothesis because our CE-MS approach does not discriminate between l-stereoisomers and d-stereoisomers.

Cystine is the oxidized dimer form of the amino acid cysteine. Researchers have also found elevated cystine levels in plasma as a consequence of a high oxidative stress or an early renal dysfunction [51]. It is tempting to speculate that the increased levels of cysteine in high glucose extracellular fluid are due to the well-known oxidative stress induced by high glucose in proximal tubular cells [17], which leads to a disulfide bond by the oxidation of two molecules of cysteine and the consequent formation of cystine. This dipeptide can inhibit tyrosine aminotransferase activity and can lead to an increased plasma tyrosine level [52,53]. This is exactly what we have observed in our study. Both cystine and tyrosine displayed higher levels in HG vs. NG groups. However, urinary excretion of tyrosine has been found to be decreased in a model of type I diabetes [44], whereas a decreased urinary excretion of tyrosine has a predictor value for the development of diabetic nephropathy in type II diabetes patients [54]. These facts suggest that the final urinary concentrations of tyrosine are not determined by the proximal tubule in diabetic patients because, in our experimental setting, the levels of tyrosine in the extracellular medium (i.e., the levels directly responsible for the urinary excretion of tyrosine) were actually increased in the HG group. This increase likely reflects a diminished uptake of tyrosine from the extracellular fluid because this amino acid is present in the formulation of the high glucose medium.

*N*-(1-Deoxy-1-fructosyl)leucine or *N*-(1-Deoxy-1-fructosyl)isoleucine and *N*-(1-Deoxy-1-fructosyl)phenylalanine were identified in our previous metabolomics study in HK-2 cells by LC-MS as well [7] and their contents in the extracellular fluid also increased. These Amadori compounds, as a consequence of producing reactive oxygen species, have an important role in the endothelial dysfunction in diabetes [55].

In both intracellular and extracellular fluids, glutamic acid was found to be an affected metabolite in high glucose-treated HK-2 cells. It had higher levels in the extracellular fluid and lower intracellular levels. Taking into account that high glucose medium does not contain glutamate, it is most likely that glutamate in this medium has been exported from the intracellular medium by an SLC7A13 transporter in exchange with cystine [56], which would explain both the increase in extracellular glutamate and the decrease in intracellular glutamate. However, additional mechanisms such as increased use of intracellular glutamic acid in other metabolic pathways and reduced import of glutamate through SLC1A1 [47] may also contribute to its lower levels in the intracellular compartment. Given that the amino acids reabsorbed in the lumen by proximal tubule are largely returned to the blood, an SLC1A1-dependent reduction in the import of the glutamate filtered by the glomeruli would contribute to the decrease in the serum levels of glutamic acid found in patients with diabetic complications [57] as well as in an animal model of type I diabetes [44]. However, specific studies should confirm this hypothesis.

## 3. Materials and Methods

### 3.1. Reagents and Solvents

All reagents employed were of analytical or MS grade. Acetonitrile, methanol, isopropanol, and formic acid were purchased from Thermo Fisher Scientific (Madrid, Spain). Water employed to prepare the running buffer was purified through a Milli-Q system from Millipore (Millipore, Madrid, Spain). Arginine, glutamic acid, pyroglutamic acid, tyrosine, alanine, histidine, cystine, proline, aspartic acid, iminodiacetic acid, 5-hydroxy-L-tryptophan, and 5,6-dihydro-5-methyluracil were acquired in Sigma Aldrich (Madrid, Spain). Methionine sulfone and tyramine used as IS and sodium hydroxide were from Sigma Aldrich while purine and hexakis(2,2,3,3-tetrafluoropropoxy)phosphazine (HP-921) solutions used as a standard mass reference were acquired from Agilent Technologies (Waldbronn, Germany).

### 3.2. HK-2 Cells Culture

HK-2 cells were acquired from the American Type Culture Collection (ATCC) (Rockville, MD, USA). The HK-2 cell line culture procedure employed was previously described by our research group [7]. HK-2 cells were maintained in DMEM/F12 supplemented with 10% fetal bovine serum, 1% penicillin/streptomycin/amphoterycin B, 1% glutamine, and 1% Insulin-Transferrine-Selenium (Thermo Fisher Scientific, Grand Island, NY, USA).

P35 culture dishes, in which the same number of cells (5 × 105 per mL) were seeded, were used to carry out the metabolomics study. Cells were plated at 90% confluence and were treated with medium DMEM-high glucose (25 mM d-glucose), normal glucose (5.5 mM d-glucose), or an osmotic control (5.5 mM d-glucose plus 19.5 mM mannitol) for 24 h. For each group, a total of seven different P35 replicates were used, where six of them were employed for the metabolomics study and the other one for cell counting and protein measurement. A hemocytometer was used to measure the number of cells via trypan blue. The Pierce BCA-200 Protein Assay Kit (ThermoFisher, Grand Island, NY, USA) was used to measure the protein content per well. In this way, 180,000, 160,000, and 130,000 cells were determined for HG, NG, and M groups, respectively, and the protein content was 0.293, 0.274, and 0.252 mg/500 µL for HG, NG, and M groups, respectively.

For the exometabolome analysis, the extracellular media were collected and stored at −80 °C until CE-MS analysis. For the endometabolome analysis, a 50 mM phosphate-buffered saline (pH 7.4) was used for washing the cells three times. Then they were trypsinized and resuspended in 1 mL of the appropriate culture medium. Lastly, they were centrifuged at 2500 rpm for 5 min and cell pellets were stored at −80 °C until CE-MS analysis.

### 3.3. Metabolite Extraction from HK-2 Cells and Culture Media

Metabolite extraction from HK-2 cells was performed following the extraction protocol previously optimized by our research group [7] with some modifications. To carry out the metabolite extraction from the HK-2 cells (intracellular fluid), 200 µL of methanol (extraction solvent) and 2 µL of each IS solution (10 mM) were added to the cell pellets obtained in Section 3.2. After vortexing for 30 s, the solution was placed in an ice bath for 5 min and, subsequently, centrifuged (14,000× *g* for 5 min at 4 °C). The supernatant was collected, and the solvent was evaporated until dryness. Then, 50 µL of 100 mM formic acid were added, vortexed for 30 s, and centrifuged at 14,000× *g* for 5 min at 4 °C. Lastly, the supernatant was placed in a glass insert to be analyzed.

The extraction of metabolites from the culture media (extracellular fluid) was performed using 100 µL of the extracellular fluid collected, as described in Section 3.2, which were mixed with 300 µL of methanol and were spiked with 2 µL of each IS solution. The solution was vortexed for 30 s, put into an ice bath for 5 min, and centrifuged at 14,000× *g* for 5 min at 4 °C. The supernatant was collected and the solvent was evaporated until dryness. The reconstitution step was the same as in the intracellular fluid.

QC samples analyzed during the metabolomic sequences were obtained mixing the same aliquot of each sample.

### 3.4. Capillary Electrophoresis–Mass Spectrometry Analysis

An Agilent 7100 CE system (Agilent Technologies, Waldbronn, Germany) coupled to a 6530 series quadrupole time-of-flight (QTOF) mass spectrometer (Agilent Technologies, Waldbronn, Germany) by means of a Jet Stream orthogonal electrospray ionization (ESI) source was employed to carry out the sample analysis. The MS system control and data acquisition were via Agilent Mass Hunter Qualitative Analysis software (B.07.00, Waldbronn, Germany). Coupling was performed using a sheath liquid interface with a CE-ESI co-axial sprayer (G1607, Agilent Technologies, Waldbronn, Germany). The sheath liquid was composed by isopropanol/water 60:40 (*v/v*) containing 0.1% formic acid as well as 0.02% (*v/v*) purine and HP-921 to produce the reference ions at *m/z* 121.0509 and *m/z* 922.0098, respectively, to allow mass accuracy monitoring. This solution was infused at 8 µL/min into the system by means of a 25 mL Gastight 1000 Series Hamilton syringe (Hamilton Robotics, Bonaduz, Switzerland) on a NE-3000 pump (New Era Pump Systems Inc., Farmingdale, NY, USA).

Separation capillary was an uncoated fused-silica capillary of 50 µm ID (362.8 µm OD) with a total length of 110 cm provided by Polymicro Technologies (Phoenix, AZ, USA). The running buffer was 1 M formic acid in water (pH 1.8) and the samples were hydrodynamically injected applying a pressure of 50 mbar for 10 s.

Before the first use, new capillaries were conditioned by flushing (1 bar) methanol, 1 M sodium hydroxide NaOH, Milli-Q water with the running buffer, for 30 min for each solution. Between runs, the capillary was conditioned with running buffer for 10 min at 1 bar. The electrophoretic separation was achieved at 25 mbar of pressure using a voltage of 30 kV for 35 min and a working temperature of 25 °C.

The ionization source conditions were: a capillary voltage of 2000 V with a nozzle voltage of 2000 V, nebulizer pressure at 10 psig, sheath gas of jet stream of 3.5 L/min at 195 °C, and drying gas of 6 L/min at 275 °C. The fragmentator (cone voltage after capillary) was set at 120 V and the skimmer and octapole voltages were 65 V at 750 V, respectively. To avoid current failures during sample injection, the nebulizer gas flow, capillary, and nozzle voltage were turned off. Then, the nebulizer gas was turned on after 0.5 min while the capillary and nozzle voltages were tuned on after 1 min. MS operated in a positive ESI mode with a mass range set at m z 70–1600 *m/z* (extended dynamic range) in full scan resolution mode at a scan rate of 2 scans per second. MS/MS analyses for metabolite identification were carried out by selecting the [M + H]^+^ ions of the metabolites as precursor ions at the given retention time with a collision energy of 20 V.

### 3.5. Metabolomic Sequence

At the beginning of the sequence, several blanks and QCs were injected in order to control the repeatability in the system. The 18 samples (six replicates per group) were randomized and a QC was introduced every six samples at the end of the sequence. The inlet vial with running buffer was changed every two injections to avoid current problems.

### 3.6. Data Treatment and Analysis

Molecular Feature Extraction tool from Mass Hunter Qualitative Analysis (B.07.00) was used to create molecular features, H^+^, Na^+^, and K^+^ were selected as possible adducts in the positive ionization mode. Molecular features filtering and alignment was performed using the tool Agilent Mass Profiler Professional (B.02.00). Migration time data was 0.5% with a window of 2.00 min both in intracellular and in extracellular fluids. Mass tolerance in the intracellular fluid was 15.0 ppm with a mass window of 2.0 mDa whereas, in the extracellular fluid, mass tolerance was 50.0 ppm with a mass window of 2.5 mDa. The migration time correction method was performed using methionine sulfone and tyramine as IS.

Multivariate statistical analysis was performed using SIMCA 14.0 (Umetrics, Umeå, Sweden). Both non-supervised analysis, principal component analysis (PCA), hierarchical cluster analysis (HCA), and the supervised analysis partial least square discriminant analysis (PLS-DA) models were carried out on log-transformed and pareto scaled data and normalized against the protein content (see Section 3.2).

The Mann-Whitney U test was used to perform the univariate statistical analysis and calculated the *p*-values, using R (http://www.R-project.org). Furthermore, the Benjamini-Hochberg false discovery rate (FDR) was employed for multiple testing correction.

### 3.7. Identification of Metabolites

The Mann-Whitney U test was allowed to select the molecular features, which changed statistically significantly. The identification of these molecular features was via a CEU Mass Mediator database from the Centre for Metabolomics and Bioanalysis (CEMBIO, Madrid, Spain) [58], which permits the simultaneous search in several databases such as HMDB (http://www.hmdb.ca/), METLIN (https://metlin.scripps.edu), LipidMaps (http://www.lipidmaps.org/), and KEGG (https://www.genome.jp/kegg/). In this database, the obtained experimental accurate mass values were searched considering an error of 30 ppm. All exogenous compounds not expected to be naturally present in biological samples (i.e., drugs among others) were discarded.

All metabolites, which were commercially available (see Section 3.1), were analyzed using the same conditions as in the metabolomics sequences, in order to obtain the migration time and MS/MS fragmentation pattern and to carry out their unequivocally identification. For those metabolites which standards were not available, a tentative identification was performed comparing the experimental MS/MS spectra obtained for each molecular feature and those predicted both in the HMDB database, CFM-ID (cfmid.wishartlab.com), and literature.

## 4. Conclusions

The present work describes the thorough development of a CE-MS method to study the metabolic differences in an in vitro model of high-glucose in a non-targeted mode. The best extraction solvent was 100% methanol in both intracellular and extracellular fluids. The optimized analysis conditions were running buffer 1 M formic acid, which applied 25 mbar of pressure during the electrophoretic separation. The sheath liquid was isopropanol/water 60:40 (*v/v*) containing 0.1% formic acid at a flow rate of 8 µL/min, and the best flow rate of the drying gas was 6 L/min. This platform adequately describes the experimental setup both for intracellular and extracellular metabolites. A total of 10 metabolites were identified. Eight metabolites were unambiguously identified in intracellular and extracellular fluids. Two metabolites were identified in extracellular fluid in a tentative manner. Most of these metabolites are amino acids but also carbohydrates and the dipeptide cystine were found. One of these metabolites, glutamic acid, was present both in intracellular and extracellular fluids. The results display the interchange existing between these two media during the metabolic process studied in this work. Overall, our results are in line with the diabetic context. This validates our metabolic strategy and highlights the importance of the CE-MS analytical platform in metabolomics analysis despite its lower use when compared to the chromatographic techniques.

## Figures and Tables

**Table 1:** R^2^X, R^2^Y, and Q^2^ parameters and the F and *p*-values of the cross validated ANOVA (CV-ANOVA) for the PLS-DA models for the two analytical sequences.

PLS-DA Models	R^2^X	R^2^Y	Q^2^	CV-ANOVA	
**Intracellular Fluid**					
HG vs. NG	0.801	0.985	0.933	F(22.5)	p(2.3 × 10^−9^)
NG vs. M	0.827	0.872	0.771	F(15.8)	p(7.7 × 10^−8^)
**Extracellular Fluid**					
HG vs. NG	0.670	0.993	0.974	F(149.2)	p(8.7 × 10^−18^)
NG vs. M	0.457	0.728	0.336	F(3.1)	p(3.3 × 10^−2^)

**Table 2:** Metabolites identified in the intracellular and extracellular analysis by the CE-MS metabolomics platform.

#	MT (min)	Molecular Formula	Identification	[M] Monoisotopic Mass (Da)	Mass Error (ppm)	Main Fragments (MS/MS)	*p*-Value **		Trend ***
							HG vs. NG	NG vs. M	
**Intracellular Fluid**									
1	9.7	C_6_H_14_N_4_O_2_	Arginine *	174.1116	0.6	130.0975 ([M + H-NH_3_-CO]^+^)	1.33 × 10^−2^	3.53 ×∙10^−5^	↓
						116.0702 ([M + H-CH_5_N_3_]^+^			
						112.0873 ([M + H-NH3-H_2_O-CO]^+^)			
						158.0950 ([M + H-NH_3_]^+^)			
2	13.5	C_5_H_9_NO_4_	Glutamic acid *	147.0531	0.7	84.0446 ([M + H-2H_2_O-CO]^+^)	7.85 ×∙10^−8^	3.81 ×∙10^−6^	↓
						56.0497 ([M + H-2H_2_O-2CO]^+^)			
3	23.8	C_5_H_7_NO_3_	Pyroglutamic acid *	129.0424	1.5	56.0493 ([M + H-H_2_O-2CO]^+^)	5.49∙× 10^−3^	2.90∙× 10^−3^	↑
						84.0443 ([M + H-H_2_O-CO] ^+^)			
**Extracellular Fluid**									
4	12.9	C_6_H_9_N_3_O_2_	Histidine *	155.0696	0.6	110.0713 ([M + H-H_2_O-CO]^+^)	1.20∙× 10^−2^	0.79	↑
						83.0602 ([M + H-H_2_O-CO-HCN]^+^)			
						93.0444 ([M + H-H_2_O-CO-NH_3_]^+^)			
5	15.8	C_3_H_7_NO_2_	Alanine *	89.0476	0.9	58.0619	5.94∙× 10^−7^	0.71	↑
						60.0784 ([M + H-CH_2_O]^+^)			
						74.0927 ([M + H-NH_2_]^+^)			
						60.9827 ([M + H-CHO]^+^)			
						72.0752 ([M + H-H_2_O]^+^)			
6	19.0	C_5_H_9_NO_2_	Proline *	115.0629	3.5	70.0638 ([M + H-H_2_O-CO]^+^)	1.11∙× 10^−4^	0.25	↑
7	19.1	C_5_H_9_NO_4_	Glutamic acid *	147.0529	2.0	84.0429 ([M + H-2H_2_O-CO]^+^)	6.38 × 10^−5^	6.62∙× 10^−2^	↑
						56.0480 ([M + H-2H_2_O-2CO]^+^)			
8	19.3	C_6_H_12_N_2_O_4_S_2_	Cystine *	240.0215	9.6	74.0216 ([M + H-C_3_H_7_NO_2_-CH_2_S_2_]])	1.00 ×∙10^−3^	0.29	↑
						120.0109 ([M + H-C_3_H_7_NO_2_S]^+^)			
						122.0247 ([M + H-C_3_H_5_NO_2_S]^+^)			
						151.9848 ([M + H-C_3_H_7_NO_2_]^+^)			
9	19.9	C_9_H_11_NO_3_	Tyrosine *	181.0731	4.4	91.0501 ([M + H-NH_3_-H_2_O-2CO]^+^)	2.28∙× 10^−2^	8.27∙× 10^−4^	↑
						136.0711 ([M + H-H_2_O-CO]^+^)			
						119.0445 ([M + H-NH_3_-H_2_O-CO]^+^)			
						123.0398 ([M + H-NH_3_-CH_2_CO]^+^)			
						95.0450 ([M + H-NH_3_-CH_2_CO-CO]^+^)			
10	24.6	C_12_H_23_NO_7_	*N*-(1-Deoxy-1-fructosyl)leucine or *N*-(1-Deoxy-1-fructosyl)isoleucine	293.1472	1.0	230.1350 ([M + H-2H_2_O-CO]^+^) 258.1301 ([M + H-2H_2_O]^+^)	8.87∙× 10^−8^	0.30	↑
						276.1404 ([M + H-H_2_O]^+^)			
11	26.3	C_15_H_21_NO_7_	*N*-(1-Deoxy-1-fructosyl)phenylalanine	327.1316	0.6	310.1226 ([M + H-H_2_O]^+^)	8.51∙× 10^−6^	0.28	↑
						292.1189 ([M + H-2H_2_O]^+^)			
						264.1281 ([M + H-H_2_O-C_2_H_5_OH]^+^)			

* Metabolites unequivocally identified with co-injection of the standard solution. ** *p*-value of Mann Whitney U test < FDR cut-off (0.040). *** ↑: The metabolite (on average) is more abundant in HG vs. NG. ↓: The metabolite (on average) is less abundant in HG vs. NG.

## Supplementary Materials

Figure S1. Comparison of the sensitivity using different flow rates of sheath liquid. Figure S2. Comparison of extraction solvents in the extracellular fluid. Figure S3. PCA normalized with IS tyramine. QC, quality control. NG, normal glucose. HG, high glucose. M, osmotic control. Figure S4. Hierarchical cluster analysis (HCA) of the two analytical sequences. Figure S5. Box-plots of the two metabolites of the intracellular fluid unequivocally identified. Figure S6. Box-plots of the seven metabolites of the extracellular fluid unequivocally and tentatively identified. Figure S7. Box-plots of the metabolite unequivocally identified in both intra and extracellular fluids. Table S1. Unknown molecular features that were statistically significant for any of the two analytical sequences.
