# Supplementary material for: A Non-Targeted Capillary Electrophoresis-Mass Spectrometry Strategy to Study Metabolic Differences in an In Vitro Model of High-Glucose Induced Changes in Human Proximal Tubular HK-2 Cells
Source: Molecules. 2020 Jan 24;25(3):512. doi: 10.3390/molecules25030512 (PMC7037647; doi:10.3390/molecules25030512)
Supplement: Supplementary file 1 [file molecules-25-00512-s001.pdf]

Supplementary information

A Non-Targeted Capillary Electrophoresis-Mass Spectrometry Strategy to Study Metabolic Differences in an *In Vitro* Model of High-Glucose Induced Changes in Human Proximal Tubular HK-2 Cells

Samuel Bernardo-Bermejo[1], Elena Sánchez-López[1], María Castro-Puyana[1,2], Selma Benito-Martínez[3,4], Francisco Javier Lucio-Cazaña[3], María Luisa Marina[1,2,*]

[1] Departamento de Química Analítica, Química Física e Ingeniería Química, Universidad de Alcalá, Ctra. Madrid-Barcelona Km. 33.600, 28871 Alcalá de Henares (Madrid), Spain.

[2] Instituto de Investigación Química Andrés M. del Río (IQAR), Universidad de Alcalá, Ctra. Madrid-Barcelona Km. 33.600, 28871 Alcalá de Henares (Madrid), Spain.

[3] Departamento de Biología de Sistemas, Universidad de Alcalá, Ctra. Madrid-Barcelona Km. 33.600, 28871 Alcalá de Henares (Madrid), Spain.

[4] Ramón y Cajal” Health Research Institute (IRYCIS), Madrid, Spain.

***Correspondence:** Departamento de Química Analítica, Química Física e Ingeniería Química, Universidad de Alcalá, Ctra. Madrid-Barcelona, Km. 33.600, 28871 Alcalá de Henares, Madrid, España.

E-mail: mluisa.marina@uah.es

Fax: +34-91 885 4971

Tel: +34-91 885 4935

Figures

Figure S1. Comparison of the sensitivity using different flow rates of sheath liquid. The figure shows the EIC of IS methionine sulfone at different flow rates: marked in red 4 µL/min, marked in black 6 µL/min and marked in green 8 µL/min.

Figure S2. Comparison of extraction solvents in the extracellular fluid. The figure shows the TCE using 100% MeOH (marked in red) and 100% ACN (marked in black).

Figure S3. PCA normalized with IS tyramine. QC, quality control; NG, normal glucose; HG, high glucose; M, osmotic control.

Figure S4. Hierarchical cluster analysis (HCA) of the two analytical sequences: A) HCA of intracellular fluid and B) HCA of extracellular fluid.

Figure S5. Box-plots of the two metabolites of the intracellular fluid unequivocally identified.

Figure S6. Box-plots of the seven metabolites of the extracellular fluid unequivocally and tentatively identified.

Figure S7. Box-plots of the metabolite unequivocally identified in both intra and extracellular fluids.

Figure S1.

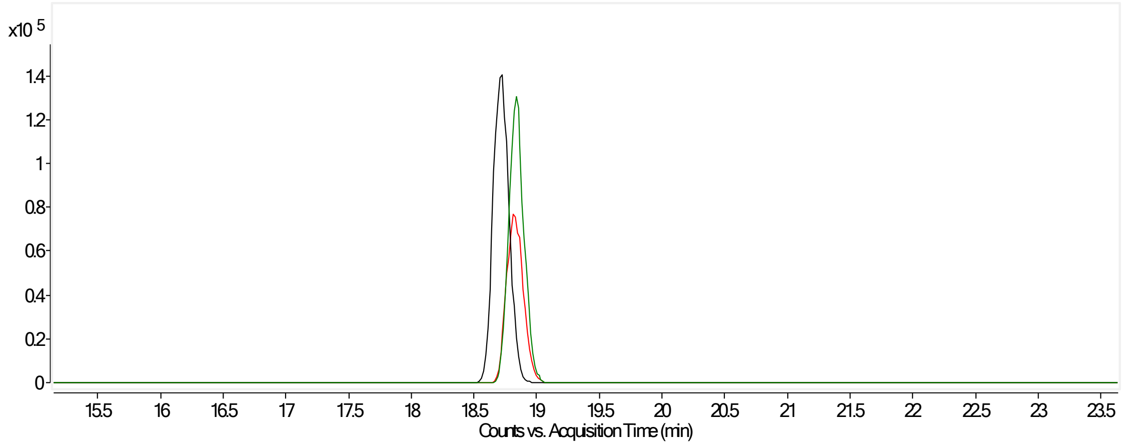
x10 5
1.4
1.2
1
0.8
0.6
0.4
0.2
0
15.5 16 16.5 17 17.5 18 18.5 19 19.5 20 20.5 21 21.5 22 22.5 23 23.5
Counts vs. Acquisition Time (min)

Figure S2.

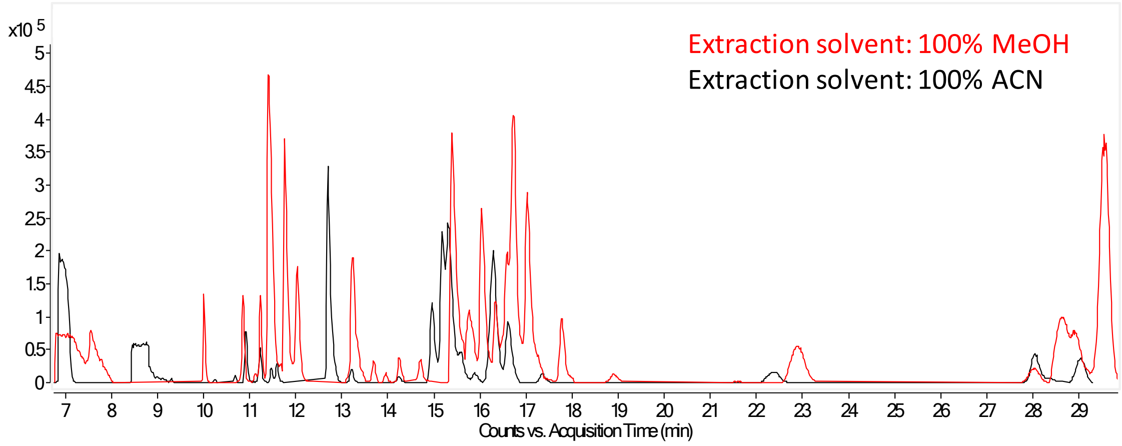
Extraction solvent: 100% MeOH
Extraction solvent: 100% ACN
x10 5
Counts vs. Acquisition Time (min)

Figura S3.

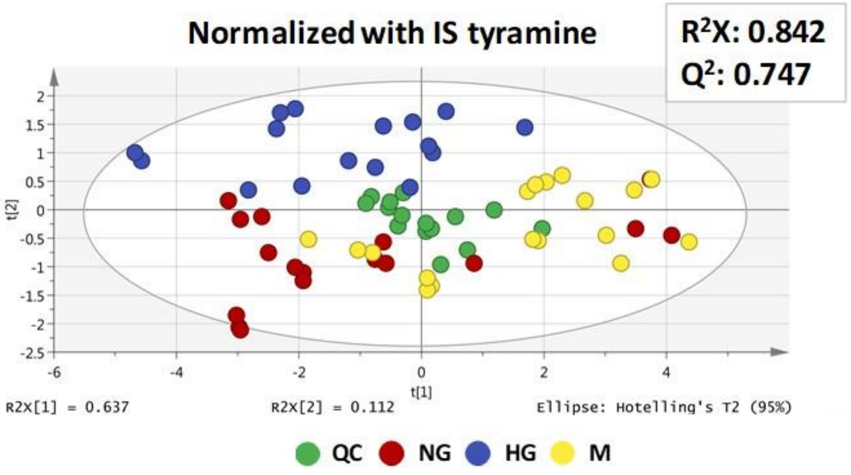

Normalized with IS tyramine
R²X: 0.842
Q²: 0.747
t[2]
t[1]
R2X[1] = 0.637
R2X[2] = 0.112
Ellipse: Hotelling's T2 (95%)
QC
NG
HG
M

Figure S4.

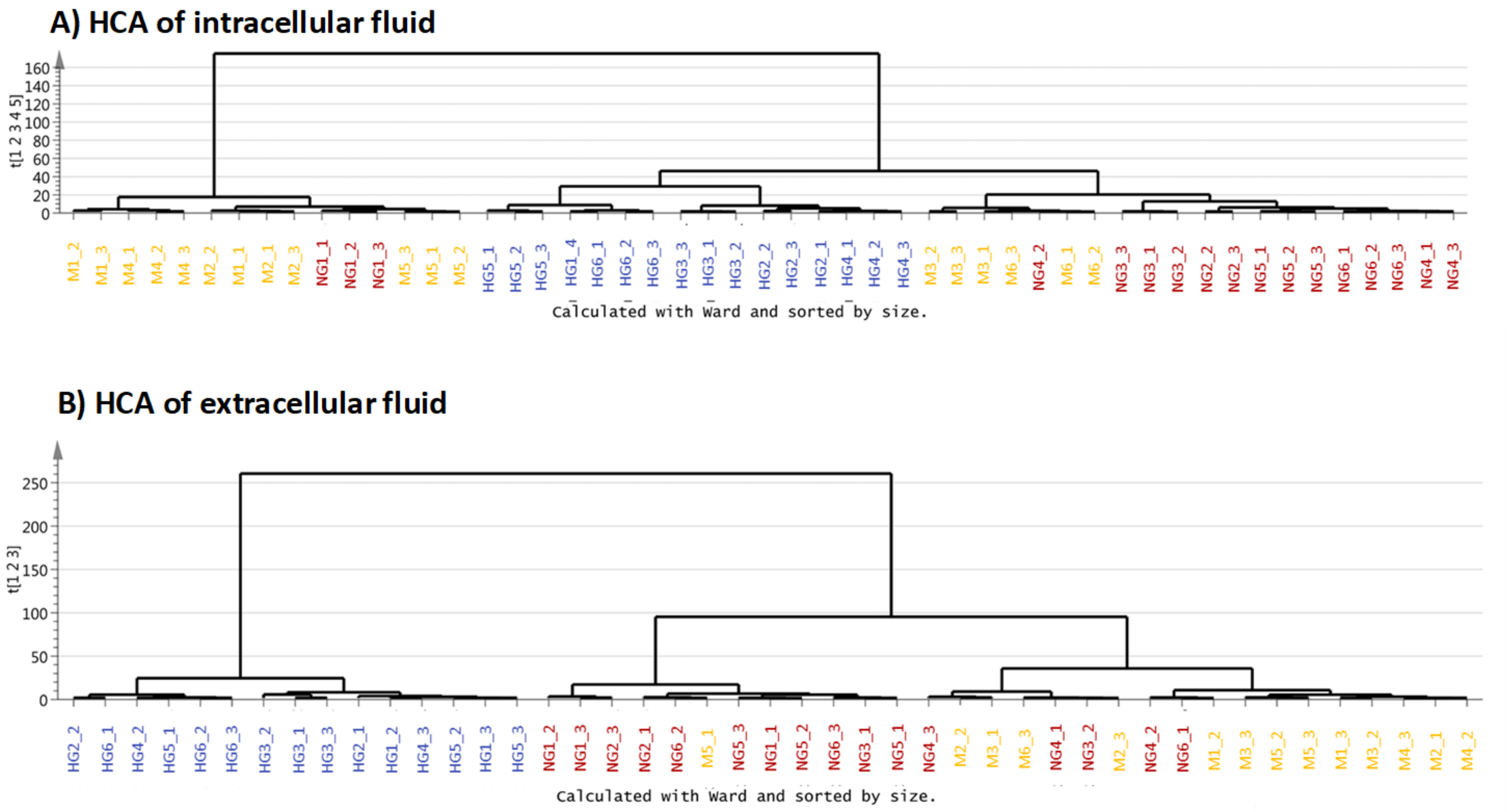
A) HCA of intracellular fluid
t[1 2 3 4 5]
160 140 120 100 80 60 40 20 0
M1_2 M1_3 M4_1 M4_2 M4_3 M2_2 M1_1 M2_1 M2_3 NG1_1 NG1_2 NG1_3 M5_3 M5_1 M5_2 HG5_1 HG5_2 HG5_3 HG1_4 HG6_1 HG6_2 HG6_3 HG3_3 HG3_1 HG3_2 HG2_2 HG2_3 HG2_1 HG4_1 HG4_2 HG4_3 M3_2 M3_3 M3_1 M6_3 NG4_2 M6_1 M6_2 NG3_3 NG3_1 NG3_2 NG2_2 NG2_3 NG5_1 NG5_2 NG5_3 NG6_1 NG6_2 NG6_3 NG4_1 NG4_3
Calculated with Ward and sorted by size.
B) HCA of extracellular fluid
t[1 2 3]
250 200 150 100 50 0
HG2_2 HG6_1 HG4_2 HG5_1 HG6_2 HG6_3 HG3_2 HG3_1 HG3_3 HG2_1 HG1_2 HG4_3 HG5_2 HG1_3 HG5_3 NG1_2 NG1_3 NG2_3 NG2_1 NG6_2 M5_1 NG5_3 NG1_1 NG5_2 NG6_3 NG3_1 NG5_1 NG4_3 M2_2 M3_1 M6_3 NG4_1 NG3_2 M2_3 NG4_2 NG6_1 M1_2 M3_3 M5_2 M5_3 M1_3 M3_2 M4_3 M2_1 M4_2
Calculated with Ward and sorted by size.

Figure S5.

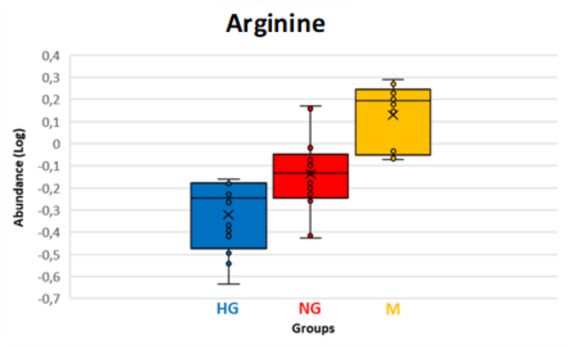
Arginine
Abundance (Log)
0,4
0,3
0,2
0,1
0
-0,1
-0,2
-0,3
-0,4
-0,5
-0,6
-0,7
HG
NG
M
Groups

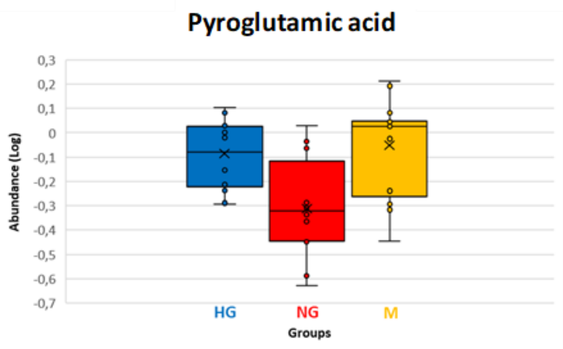
Pyroglutamic acid
Abundance (Log)
0,3
0,2
0,1
0
-0,1
-0,2
-0,3
-0,4
-0,5
-0,6
-0,7
HG
NG
M
Groups

Figure S6.

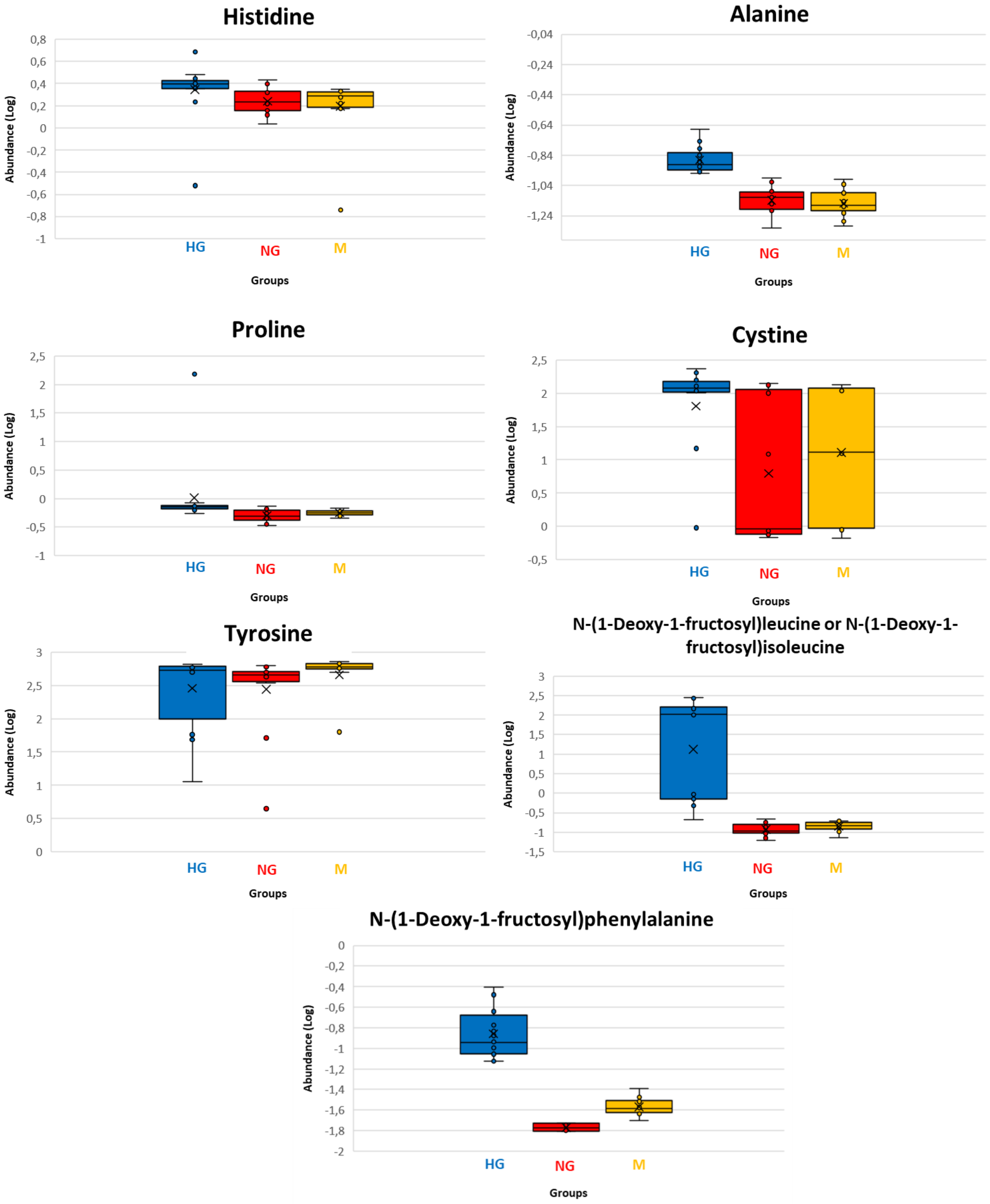
Histidine
Abundance (Log)
0,8
0,6
0,4
0,2
0
-0,2
-0,4
-0,6
-0,8
-1
HG
NG
M
Groups
Alanine
Abundance (Log)
-0,04
-0,24
-0,44
-0,64
-0,84
-1,04
-1,24
HG
NG
M
Groups
Proline
Abundance (Log)
2,5
2
1,5
1
0,5
0
-0,5
-1
HG
NG
M
Groups
Cystine
Abundance (Log)
2,5
2
1,5
1
0,5
0
-0,5
HG
NG
M
Groups
Tyrosine
Abundance (Log)
3
2,5
2
1,5
1
0,5
0
HG
NG
M
Groups
N-(1-Deoxy-1-fructosyl)leucine or N-(1-Deoxy-1-fructosyl)isoleucine
Abundance (Log)
3
2,5
2
1,5
1
0,5
0
-0,5
-1
-1,5
HG
NG
M
Groups
N-(1-Deoxy-1-fructosyl)phenylalanine
Abundance (Log)
0
-0,2
-0,4
-0,6
-0,8
-1
-1,2
-1,4
-1,6
-1,8
-2
HG
NG
M
Groups

Figure S7.

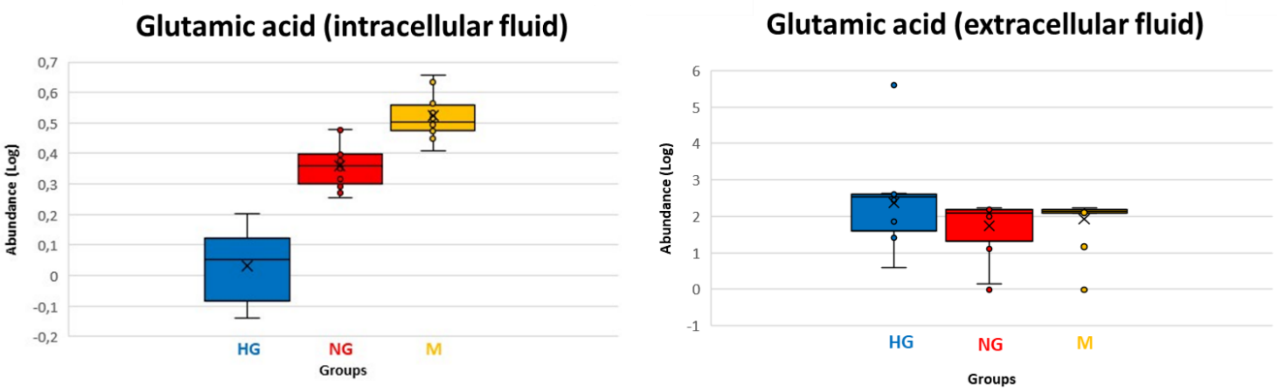
Glutamic acid (intracellular fluid)
0,7
0,6
0,5
0,4
0,3
0,2
0,1
0
-0,1
-0,2
Abundance (Log)
HG
NG
M
Groups
Glutamic acid (extracellular fluid)
6
5
4
3
2
1
0
-1
Abundance (Log)
HG
NG
M
Groups

Table S1. Unknown molecular features which were statistically significant for any of the two analytical sequences

#	MT (min)	Monoisotopic mass (Da)	Main fragments	p-value*		Trend**
				HG *vs* NG	NG *vs* M	
**Intracellular fluid**						
1	10.9	503.2651	-	1.61×10^{-2}	1.1×10^{-2}	↓
2	10.9	475.2328	-	1.34×10^{-2}	1.73×10^{-4}	↓
3	13.2	299.1356	121.0635, 282.1352	2.32×10^{-7}	8.92×10^{-2}	↑
4	13.5	775.4005	-	3.88×10^{-2}	1.82×10^{-4}	↓
5	13.5	575.2941	-	1.01×10^{-7}	6.93×10^{-8}	↓
6	13.5	547.2623	-	1.35×10^{-7}	9.10×10^{-8}	↓
7	14.5	609.2809	-	1.08×10^{-3}	0.14	↓
8	14.5	581.2505	-	9.39×10^{-4}	2.88×10^{-2}	↓
9	14.5	809.3868	-	6.34×10^{-3}	0.61	↓
10	18.5	308.1188	-	1.78×10^{-4}	3.94×10^{-4}	↑
**Extracellular fluid**						
11	10.3	203.9495	90.9754, 84.9587	3.38×10^{-2}	1.55×10^{-2}	↑
12	11.9	262.1397	246.1212, 221.1233, 204.0974	5.56×10^{-6}	0.34	↓
13	12.9	109.0640	74.0970, 94.0400	3.22×10^{-3}	0.67	↑
14	13.0	767.3479	427.1522, 327.0499, 191.0769	2.17×10^{-3}	0.25	↓
15	13.1	712.3085	381.2971, 226.9525	8.32×10^{-8}	0.16	↑
16	13.8	774.3469	482.2129	1.46×10^{-5}	0.99	↑
17	15.3	308.1535	128.0627, 211.0961, 84.0734, 225.1129	5.12×10^{-8}	0.14	↑
18	15.4	128.0555	84.0790, 121.0623	2.18×10^{-6}	0.63	↓
19	15.8	491.3196	226.9489, 90.9748, 158.9617, 381.2956	3.51×10^{-4}	0.11	↑
20	15.8	447.3559	90.9737, 381.2967	4.25×10^{-6}	0.32	↑
21	15.8	419.3233	90.9745, 210.0451, 308.1164	1.86×10^{-3}	0.89	↑
22	16.4	308.1582	191.0708, 90.9734	4.75×10^{-8}	0.19	↑
23	16.5	573.3849	226.9518, 242.9260	6.62×10^{-5}	9.54×10^{-2}	↓
24	16.5	193.0949	84.9565, 78.9930	3.06×10^{-4}	0.17	↓
25	16.6	297.0451	-	6.16×10^{-5}	6.52×10^{-2}	↑
26	16.7	336.1645	90.9740, 158.9606	7.83×10^{-8}	17.50×10^{-2}	↑
27	17.2	266.1380	149.0218, 122.0675, 90.9748	1.17×10^{-4}	0.33	↓
28	17.3	317.1221	143.0272, 202.0770, 176.0629, 164.0239	1.07×10^{-2}	0.00	↑
29	19.2	527.3422	314.0307, 120.0033, 391.0416, 217.0165	2.68×10^{-3}	0.38	↑
30	20.3	133.0373	-	9.38×10^{-3}	0.74	↑
31	23.1	220.0826	-	5.30×10^{-4}	0.26	↑

32	24.18	225.0746	78.9956, 106.9899, 90.9746, 116.9731136.0761	5.93×10^{-5}	0.34	↓
33	24.80	279.1324	90.9743	9.60×10^{-3}	0.74	↑
34	25.10	296.0501	176.0593, 120.0036	2.59×10^{-6}	0.37	↑
35	26.28	343.1268	90.9744, 57.0682, 158.9627	1.96×10^{-5}	0.23	↑
36	27.26	574.1223	226.9493, 158.9635	1.14×10^{-4}	0.13	↓

*p-value of Mann Whitney U test < FDR cut-off (0.040).

**↑: The metabolite (on average) is more abundant in HG vs NG; ↓: The metabolite (on average) is less abundant in HG vs NG.
